# 电磁脉冲对A549细胞分泌外泌体的影响

**DOI:** 10.3779/j.issn.1009-3419.2024.106.34

**Published:** 2024-12-20

**Authors:** Qingxia HOU, Yingmei WANG, Meng CAO, Jiangzheng LIU, Deqin KONG, Qian ZHANG, Weihua YU, Guangzhou AN

**Affiliations:** ^1^710032 西安，空军军医大学第一附属医院病理科; ^1^Department of Pathology, the First Affiliated Hospital of Air Force Medical University; ^2^空军军医大学军事预防医学系毒理学与防化医学教研室; ^2^Department of Toxicology and Chemical Defense Medicine, Faculty of Preventive Medicine, Air Force Medical University; ^3^特殊作业环境危害评估与防治教育部重点实验室; ^3^Ministry of Education Key Laboratory of Hazard Assessment and Control in Special Operational Environment, Xi’an 710032, China

**Keywords:** 肺肿瘤, A549细胞, 外泌体, 电磁脉冲, miRNA, Lung neoplasms, A549 cells, Exosomes, Electromagnetic pulses, miRNA

## Abstract

**背景与目的:**

大量研究提示电磁脉冲（electromagnetic pulses, EMP）在肿瘤治疗方面具有靶向性强、副作用少和治疗费用低等优点，但其用于肿瘤治疗的最佳参数以及EMP与肿瘤外泌体之间的关系至今仍不清楚。本研究旨在明确不同参数EMP对人非小细胞肺癌A549细胞分泌外泌体数量和内含物微小RNA（microRNA, miRNA）的影响，为EMP临床应用和相关研究提供参考。

**方法:**

将A549细胞随机分为对照组和不同强度EMP辐照组，辐照组细胞分别接受场强为400、600和800 kV/m EMP辐照，EMP脉冲数为2000个，重复频率为20 Hz，脉宽为120 ns，每天辐照1次，连续辐照3 d。辐照结束后，收集并鉴定外泌体，台盼蓝染色检测活细胞数量变化，纳米颗粒追踪分析（nanoparticle tracking analysis, NTA）检测外泌体浓度变化，miRNA测序检测外泌体内miRNA丰度变化。

**结果:**

与对照组相比，EMP辐照组A549细胞的形态和数量未见明显改变，但400或800 kV/m EMP辐照组外泌体数量明显减少（*P*<0.05），600 kV/m EMP辐照组外泌体数量明显增多（*P*<0.05）。各个EMP辐照组与对照组相比，外泌体miRNAs的表达丰度明显不同（*P*<0.05），其差异丰度miRNAs靶基因富集在不同的细胞信号通路。

**结论:**

实验条件下EMP辐照可明显改变A549细胞分泌外泌体的数量和miRNAs的丰度，可进一步影响肿瘤外泌体功能。

## Abstract (en)

**Background and objective:**

Numerous researches indicated that electromagnetic pulses (EMP) possessed advantages such as strong targeting, minimal side-effects and low treatment cost in tumor therapy, but its optimum parameters for treatment and the relationship between EMP and tumor-derived exosomes remains unclear. This study aims to clarify the effects of EMP with different parameters on the quantity and miRNA (microRNA) of exosomes secreted by human non-small cell lung cancer A549 cells, providing beneficial reference for the clinical application of EMP and related research.

**Methods:**

A549 cells were randomly divided into control group and different EMP radiation groups with respective intensity of 400, 600 and 800 kV/m. EMP was performed with 2000 pulses once, 20 Hz of repetition frequency and 120 ns of pulse width. A549 cells were radiated once per day for continuous 3 days. After radiation, exosomes were collected and identified; cell number was measured by trypan blue staining; the concentration of exosomes was measured by nanoparticle tracking analysis (NTA); the abundance of miRNAs was determined by miRNA sequencing.

**Results:**

Compared with control group, the morphology and cell viability of A549 cells in radiation group was not different, but the quantity of exosomes in 400 or 800 kV/m radiation group was significantly decreased (*P*<0.05), in contrast with obvious increase in 600 kV/m radiation group (*P*<0.05). The abundance of exosomal miRNAs between control group and each EMP group was obviously different (*P*<0.05) and target genes of differentially abundant miRNAs enriched in different pathways.

**Conclusion:**

Under the experimental condition, the quantity and miRNA abundance of exosomes could be changed by EMP radiation, which could further influence the function of tumor-derived exosomes.

肺癌是一种高发病率和死亡率的恶性疾病，我国肺癌粗发病率高达57.3/10万，死亡率为45.9/10万，位居所有癌症之首，严重威胁着国人的生命健康^[1]^。目前，对于肺癌的治疗主要根据肺癌的病理类型和肺癌分期选择相应的治疗策略，治疗方法包括手术、放疗、化疗、分子靶向治疗和免疫治疗等，但对于大量的晚期肺癌患者仍然缺乏理想的治疗方法，新的治疗靶点和治疗策略正在不断探索当中。体内外研究^[2⇓⇓-5]^发现，电磁脉冲（electromagnetic pulses, EMP）可通过抑制肿瘤细胞增殖或诱导肿瘤细胞凋亡等方式延缓肿瘤的发展和提高肿瘤患者的整体生存率，在肿瘤治疗方面具有潜在的应用价值，具有靶向性强、副作用少和治疗费用低等优势，但关于EMP治疗肿瘤的理想参数和作用机制方面仍有待探索。

肿瘤外泌体是肿瘤细胞产生和释放的直径在30-200 nm的杯状或盘状囊泡，囊泡内含有微小RNA（microRNA, miRNA）、蛋白分子和DNA片段等功能分子，扮演着细胞间通讯或信号转导的重要角色，参与了从肿瘤细胞产生到转移的整个过程。大量文献^[6,7]^报道证实，肿瘤外泌体在肿瘤上皮间充质转化和靶向转移中发挥着关键作用。同时，肿瘤外泌体还可提高肿瘤细胞侵袭能力、促进肿瘤细胞增殖、抑制肿瘤细胞凋亡或增强肿瘤细胞的治疗抵抗^[8,9]^。研究^[10,11]^表明，抑制肿瘤外泌体功能分子表达如miRNA表达可有效抑制肿瘤进展。但EMP能否通过抑制肿瘤外泌体功能分子的表达抑制肿瘤的发展至今未见报道，关于EMP与肿瘤外泌体的关系至今仍不清楚。因此，本研究以A549细胞为研究对象，主要通过纳米颗粒追踪分析（nanoparticle tracking analysis, NTA）和外泌体组学等方法手段探讨EMP对肿瘤外泌体浓度的影响及其与肿瘤外泌体功能分子的关系，为明确EMP能否影响肿瘤外泌体的生成和功能分子的表达以及肿瘤外泌体的功能提供参考。

## 1 材料与方法

### 1.1 主要仪器与试剂

超净工作台（美国TermoFisher公司），细胞培养箱（美国TermoFisher公司），超速离心机（美国Beckman公司），倒置显微镜（德国Leica公司），透射电镜（美国FEI公司），纳米颗粒跟踪分析仪（德国Particle Metrix公司），Illumina NovaSeq 6000测序平台（美国Illumina公司），TapeStation 4200（美国Agilent公司），Qubit 3.0 Fluorometer（美国TermoFisher公司）；F12K培养基（美国Invitrogen公司），无外泌体胎牛血清（以色列Bioind公司），常规胎牛血清、0.25%胰酶溶液、磷酸盐缓冲液（phosphate-buffered saline, PBS）、Dulbecco氏PBS（Dulbecco's PBS, DPBS）和青-链霉素均购自美国Gibco公司，0.4%台盼蓝染液（中国索莱宝公司），1.5%磷钨酸（中国大茂化学试剂厂），miRNA提取试剂盒（德国Qiagen公司），QIAseq miRNA文库试剂盒（德国Qiagen公司）。

### 1.2 细胞培养与分组

人肺腺癌A549细胞购自美国ATCC公司，细胞培养液为含10%胎牛血清和1%青链霉素的F12K完全培养液，A549细胞置于温度为37 ^o^C和5% CO_2_浓度的细胞孵箱中常规培养。细胞密度达到80%-90%时进行细胞传代，每3 d传代一次。EMP辐照前，细胞密度为50%-60%，细胞培养液更换为无外泌体细胞培养液，将细胞随机分为对照组以及400、600和800 kV/m的EMP强度辐照组，每组10瓶细胞。

### 1.3 EMP辐照

EMP辐照系统由信号源、辐照腔、脉冲负载和稳压器组成。辐照腔最大体积为50 cm×50 cm×50 cm，可通过调节顶板与底板之间的距离对辐照强度进行调节。EMP辐照前从细胞培养瓶吸出细胞培养液，只保留少许细胞培养液，当辐照进行时辐照腔内EMP强度是均一的。本研究根据文献^[2]^报道和EMP辐照系统可产生的最大强度选择EMP辐照参数，其中，EMP重复频率为20 Hz，单次辐照脉冲数为2000脉冲，辐照强度分别为400、600和800 kV/m，脉宽为120 ns，上升前沿为40 ns，辐照参数由示波器实时监测，以确保严格按照辐照参数辐照细胞。辐照结束后将细胞培养液重新添回培养瓶。辐照组细胞每日EMP辐照1次，连续辐照3 d。对照组细胞在相同实验条件下不进行EMP辐照。

### 1.4 提取外泌体

利用差速离心法提取外泌体。EMP辐照结束后A549细胞继续培养4 h，然后收集细胞培养液。细胞培养液2000 g离心10 min收集上清液，上清液1000 g离心30 min后将上清转移至超速离心管中，110,000 g离心75 min，弃上清。离心管沉淀用1 mL PBS重悬，然后利用0.22 μm膜过滤。过滤后样本110,000 g再次离心75 min，弃上清。最后，离心管沉淀用1 mL PBS重悬，开展后续实验或置于-80 ^o^C冰箱中保存。

### 1.5 透射电镜观察外泌体形态

提取的外泌体用PBS稀释20倍后，将10 μL外泌体溶液滴加到铜网上，室温吸附5 min，用滤纸小心吸除多余液体。然后向铜网上滴加10 μL的1.5%磷钨酸溶液（pH为6.5），室温下对外泌体染色2 min，用滤纸小心吸除多余的染液，铜网在室温下晾干，上机检测。

### 1.6 NTA检测外泌体粒径和浓度

根据纳米颗粒跟踪分析仪说明书进行NTA分析。打开ZetaView软件，样品池清洁度和空白对照样品检测完成后设置相机参数，Min Bright、Min Size、Max Size、Sensitivity、Shutter分别设置为20、5、1000、70、70。颗粒稳定后设置软件测量界面参数，其中Cycles设置为3。然后设置实验参数，将样品用PBS稀释2000倍后上机进行检测。检测完成后对样本池进行清洗。

### 1.7 台盼蓝法检测活细胞数量

EMP辐照前每个培养瓶接种相同细胞数量的A549细胞。EMP辐照结束后用0.25%胰酶溶液消化细胞，制备细胞悬液。将细胞悬液与0.4%台盼蓝溶液以9:1比例混合均匀，在倒置显微镜下进行细胞计数，其中活细胞呈无色透明状，而死细胞呈现明显的蓝色。对活细胞计数3次取平均值。

### 1.8 外泌体miRNA测序和定量

提取后的外泌体用干冰冷冻邮寄给上海华盈生物医药科技有限公司进行外泌体miRNA测序。采用miRNA提取试剂盒并根据厂商操作说明书提取样本miRNA并纯化，然后分别利用TapeStation 4200和Qubit 3.0 Fluorometer对样本进行电泳质检和定量质检。利用QIAseq miRNA文库试剂盒并根据厂商操作说明书构建miRNA文库，并利用TapeStation 4200和Qubit 3.0 Fluorometer进行质检。利用Illumina NovaSeq 6000平台对外泌体样本进行miRNA测序。利用Cutadapt软件处理测序接头，然后利用perl5软件处理掉唯一分子标识符（unique molecular identifiers, UMIs）<12 bp的reads和没有UMI的reads并识别UMIs，最后利用bowtie软件比对到miRBase数据库的reads进行miRNA定量。

### 1.9 差异表达基因鉴定和生信分析

使用R包edgeR对得到的表达UMI counts矩阵根据分组信息进行表达差异计算，通过Benjamini和Hochberg方法对P值进行调整，以控制假阳性率。调整后的*P*<0.05时，认为存在统计学差异，筛选出差异的miRNAs利用R软件绘制火山图。利用miRTarBase数据库对差异的miRNA进行靶基因预测注释，并利用DAVID在线分析平台（https://david.ncifcrf.gov/）对靶基因进行基因本体论（Gene Ontology, GO）和京都基因和基因组数据库（Kyoto Encyclopedia of Genes and Genomes, KEGG）分析。GO分析包括生物过程（biological processes, BP）、细胞组分（cellular components, CC）和分子功能（molecular functions, MF）三个条目。GO和KEGG分析结果分别利用R软件包“GOplot”和“ggplot2”可视化，*P*<0.05认为差异具有统计学意义。

### 1.10 统计分析

每个实验在相同条件下独立重复3次，利用SPSS 26.0软件对实验数据进行统计分析。两组样本间均数的比较采用t检验，以*P*<0.05为差异有统计学意义。

## 2 结果

### 2.1 EMP对A549细胞形态的影响

倒置显微镜下观察发现，与对照组相比，400、600和800 kV/m EMP辐照组A549细胞的形态未发生明显变化（图1）。

**图 1:**
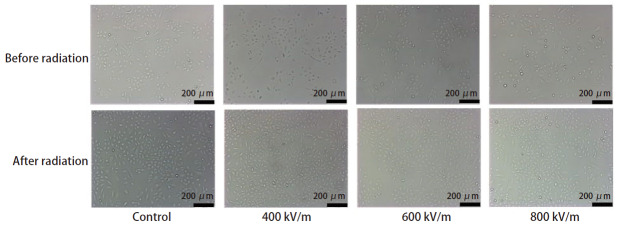
EMP辐照后A549细胞形态的变化。所示图片为各组代表性图片，比例尺为200 μm。

### 2.2 EMP对A549细胞数量的影响

台盼蓝染色结果显示，EMP辐照结束后，与对照组相比，400、600和800 kV/m EMP辐照组A549活细胞数量未发生明显变化（图2）。

**图 2:**
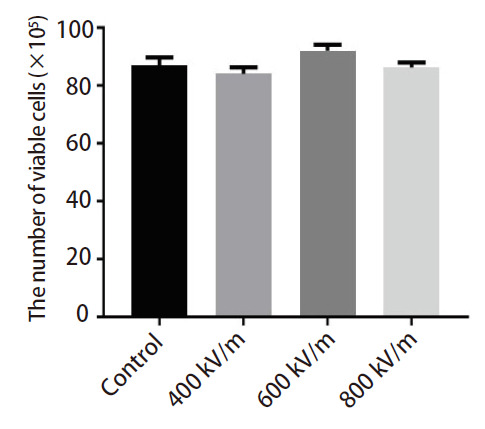
EMP辐照后A549活细胞数量的变化。所示数据为3个独立实验的均值±标准误。

### 2.3 外泌体鉴定结果

电镜下观察发现，提取的外泌体呈现典型的杯状结构，长径在30-200 nm（图3A）。NTA检测结果显示，外泌体的粒径小于200 nm（图3B）。

**图 3:**
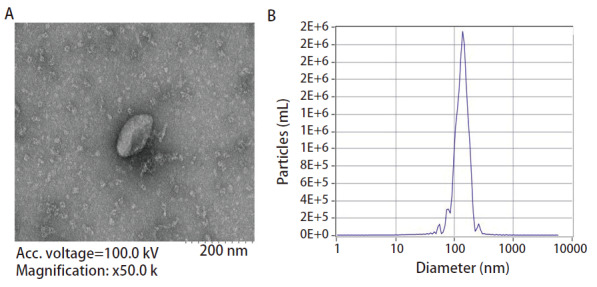
外泌体鉴定结果。A：电镜下观察外泌体形态，比例尺为200 nm；B：NTA检测外泌体粒径大小。

### 2.4 EMP对A549细胞分泌外泌体数量的影响

因各组A549细胞的数量和培养上清体积是相同的，所以细胞上清外泌体浓度可反映A549细胞分泌外泌体的数量。NTA检测结果（图4）发现，与对照组相比，400和800 kV/m EMP辐照组外泌体浓度明显下降（*P*<0.05），600 kV/m EMP辐照组外泌体浓度明显升高（*P*<0.05）；与400或800 kV/m EMP辐照组相比，600 kV/m EMP辐照组外泌体浓度明显升高（*P*<0.05）；与400 kV/m EMP辐照组相比，800 kV/m EMP辐照组外泌体浓度未发生明显变化。

**图 4:**
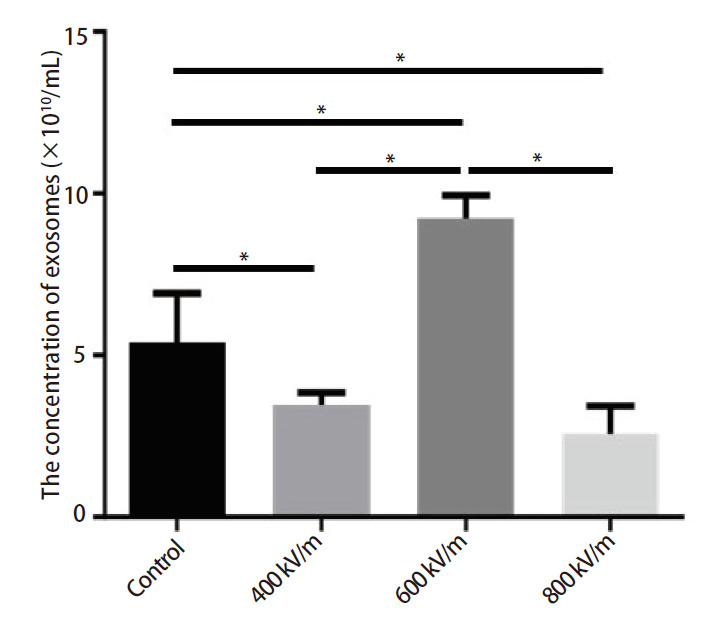
EMP辐照后外泌体浓度的变化。所示数据为3个独立实验的均值±标准误。*P<0.05。

### 2.5 EMP对A549细胞外泌体miRNAs丰度的影响

外泌体miRNA测序结果显示，每组共鉴定出2656个miRNAs，400 kV/m组与对照组外泌体有305个miRNAs丰度存在明显差异，其中丰度增加的有106个miRNAs，丰度降低的有199个miRNAs（图5A，*P*<0.05）；600 kV/m组与对照组外泌体有99个miRNAs丰度存在明显差异，其中丰度增加的有39个miRNAs，丰度降低的有60个miRNAs（图5B，*P*<0.05）；800 kV/m组与对照组外泌体有293个miRNAs丰度存在明显差异，其中丰度增加的有61个miRNAs，丰度降低的有232个miRNAs（图5C，*P*<0.05）。韦恩图显示，共有31个miRNAs在3个辐照组中的丰度与对照组相比均显著升高（图5D，*P*<0.05）。

**图 5:**
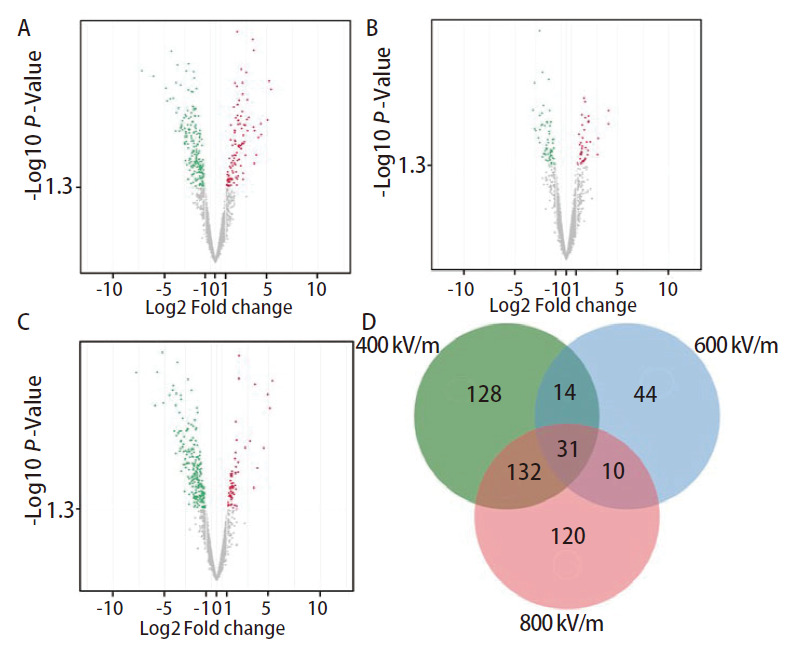
EMP辐照后A549细胞外泌体miRNA丰度的差异。A-C：400 kV/m组、600 kV/m组、800 kV/m组分别与对照组A549细胞外泌体miRNA丰度的差异。绿点代表EMP辐照后丰度降低的miRNA（*P*<0.05）；红点代表EMP辐照后丰度增加的miRNA（*P*<0.05）。D: 丰度差异miRNA的韦恩图。

### 2.6 生信分析结果

GO分析结果显示，400 kV/m组与对照组差异丰度miRNAs靶基因富集在细胞黏附分子结合、转录共激活因子活性和钙黏蛋白结合等分子功能条目，聚焦黏附、细胞基质黏附连接和细胞连接等细胞组分，组蛋白修饰、共价染色质修饰和肽基-赖氨酸修饰等生物过程（图6A）；600 kV/m组与对照组差异丰度miRNAs靶基因富集在钙黏蛋白结合、细胞黏附分子结合和蛋白磷酸酶结合等分子功能条目，聚焦黏附、细胞基质黏附连接和细胞连接等细胞组分，蛋白稳定性调节、蛋白稳定性和mRNA处理等生物过程（图6B）；800 kV/m组与对照组差异丰度miRNAs靶基因富集在细胞黏附分子结合、钙黏蛋白结合和转录共激活因子活性等分子功能条目，聚焦黏附、细胞基质黏附连接和细胞连接等细胞组分，细胞分解代谢正向调节、染色体组织调节和mRNA分解代谢等细胞过程（图6C）。KEGG分析结果显示，400 kV/m组与对照组差异丰度miRNAs靶基因富集在肿瘤蛋白多糖、Hippo、MAPK和ERBB等细胞信号通路（图7A），600 kV/m组与对照组差异丰度miRNAs靶基因富集在肿瘤蛋白多糖、Hippo、MAPK和ERBB等细胞信号通路（图7B），800 kV/m组与对照组差异丰度miRNAs靶基因富集在细胞衰老、Hippo、MAPK和ERBB等细胞信号通路（图7C）。

**图 6:**
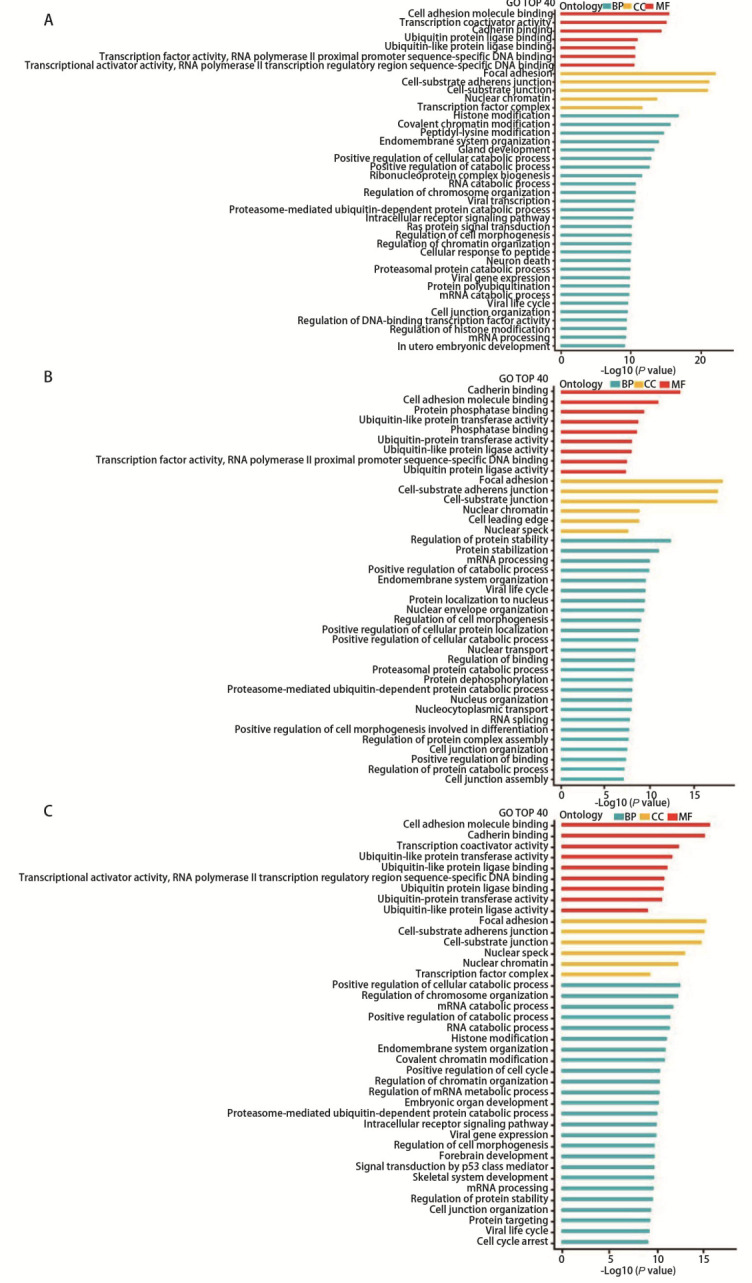
EMP辐照A549细胞后外泌体差异丰度miRNAs靶基因GO分析结果。A-C：400 kV/m组、600 kV/m组、800 kV/m组分别与对照组外泌体差异丰度miRNAs靶基因的GO分析结果。

**图 7:**
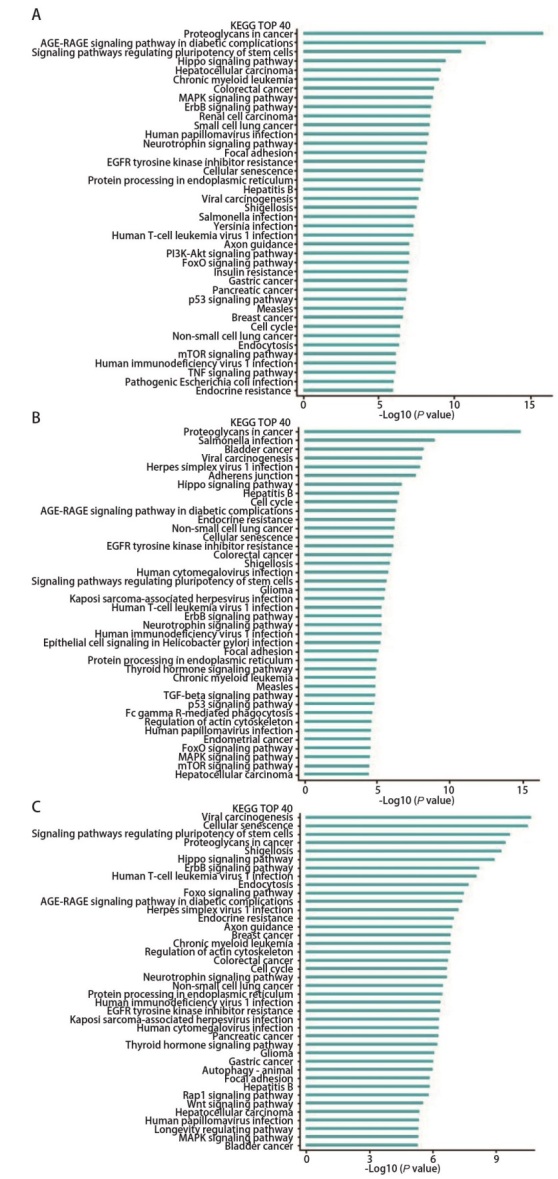
EMP辐照A549细胞后外泌体差异丰度miRNAs靶基因KEGG分析结果。A-C：400 kV/m组、600 kV/m组、800 kV/m组分别与对照组外泌体差异丰度miRNAs靶基因的KEGG分析结果。

## 3 讨论

本研究首次探讨了不同参数EMP对肿瘤外泌体生成和功能分子表达的影响，发现实验条件下的EMP虽然不能改变A549细胞的形态，也不能直接消除A549细胞或影响A549细胞数量，但可明显影响外泌体的浓度和多种miRNAs分子的丰度。一些研究报道EMP可靶向消除肿瘤细胞和减少肿瘤细胞的数量，如Pantelis课题组^[12]^发现，磁通量为0.011 T的EMP同时辐照乳腺癌上皮细胞和正常的成纤维细胞，可降低乳腺癌细胞的活力，但对成纤维细胞的活力无影响。该研究选择的EMP辐照参数、辐照条件和辐照对象与本研究不同，这可能是造成结果不一致的主要原因。在一些高强度和陡脉冲的EMP辐照下，一些肿瘤细胞膜甚至可发生电穿孔，引起肿瘤细胞的死亡^[13]^。本研究选择相对安全的EMP辐照参数，探讨低强度下EMP对肿瘤细胞和肿瘤外泌体的影响。有研究^[14,15]^提示，低强度EMP具有显著的非热生物学效应，但产生的生物学效应和具体的实验条件和研究对象有关。

尽管实验条件下EMP不能直接消除A549细胞，但可能会通过外泌体途径影响肿瘤的进展。肿瘤外泌体与肿瘤的转移等恶性行为密切相关，肿瘤外泌体浓度和内含物丰度的改变可影响肿瘤的进展。利用外泌体生成抑制剂可降低A549细胞分泌肿瘤外泌体的浓度，进而减轻肿瘤外泌体介导的生物学效应^[16,17]^。本研究发现，EMP同样可以影响A549细胞肿瘤外泌体的分泌，400和800 kV/m强度EMP可明显抑制A549细胞分泌肿瘤外泌体，但600 kV/m EMP却可促进A549细胞分泌肿瘤外泌体，提示其效应和特定的辐照强度密切相关，并非简单的呈现“剂量-效应”线性规律，这可能与电磁辐射的“窗效应”及细胞特定信号通路激活有关^[18,19]^。本研究在选择提取外泌体时间点时，选择了辐照后4 h作为观察时间，主要考虑随着时间延长，A549细胞分泌的外泌体可能更多地反映细胞恢复状态，而不是EMP直接作用的结果。尽管未设置时间梯度，但通过NTA验证了4 h收集的外泌体浓度符合实验需求，为后续实验提供了可靠基础。

为明确EMP对肿瘤外泌体内含物的影响，本研究对肿瘤外泌体miRNAs测序并进行生信分析，发现不同强度的EMP均可影响肿瘤外泌体的miRNAs丰度；同时，生信分析结果提示，EMP辐照后肿瘤外泌体差异丰度miRNAs靶基因富集在不同的GO条目和细胞信号通路，且与辐照强度密切相关，提示不同强度EMP辐照后A549细胞分泌的肿瘤外泌体对受体细胞可能有不同的影响。值得注意的是，不同强度EMP辐照后A549细胞分泌的肿瘤外泌体差异丰度miRNAs靶基因均富集到“细胞黏附分子结合”分子功能条目，而细胞黏附与肿瘤转移密切相关，提示实验条件下不同强度EMP辐照后A549细胞分泌的肿瘤外泌体可影响肿瘤的侵袭转移，进而影响肿瘤的进展。此外，在KEGG分析结果中均富集到与肿瘤转移和进展相关的细胞信号通路，如不同EMP辐照强度下均富集到Hippo、MAPK和ERBB细胞信号通路，而这些细胞信号通路的改变与肿瘤的进展密切相关^[20⇓-22]^。需要说明的是，本研究中miRNA丰度差异的结果来源于外泌体miRNA测序分析，在后续研究中课题组计划结合分子功能分析和定量聚合酶链式反应（quantitative polymerase chain reaction, qPCR）验证实验，进一步筛选差异表达miRNA分子，明确EMP调控的具体靶分子及其作用机制。

EMP与外泌体关系的报道较少，主要集中在EMP对外泌体介导的骨组织修复的研究。国内学者研究^[23,24]^发现，1 mT EMP辐照后间充质干细胞分泌的外泌体抑制软骨细胞凋亡效应显著增强，1 mT EMP也可促进间充质细胞来源的外泌体介导的骨关节炎软骨再生，其中，75 Hz的EMP的干预效果优于15或45 Hz的EMP。这些研究结果提示，EMP可影响间充质干细胞分泌外泌体的浓度或内含物的组成，与本研究的实验结果一致。本研究通过NTA和miRNA测序等技术，初步探讨了EMP对肿瘤外泌体生成及其内含物的影响，填补了EMP与肿瘤外泌体关系研究的空白。研究结果提示，EMP可通过调控肿瘤外泌体的分泌及其内含物的组成，间接影响肿瘤细胞的行为和功能，为EMP在肿瘤治疗中的潜在应用提供了理论基础和实验依据。在后续研究中，课题组计划进一步深入探索EMP对肿瘤细胞行为和功能的影响，明确其在调控肿瘤发生、发展及转移中的分子机制，从而为EMP的临床转化和精准治疗提供可靠的数据支持。
